# Effect of preemptive analgesia with ibuprofen in the control of postoperative pain in dental implant surgeries: A randomized, triple-blind controlled clinical trial

**DOI:** 10.4317/medoral.56171

**Published:** 2020-01-01

**Authors:** Gustavo-Mattos Pereira, Luís-Otávio-Miranda Cota, Rafael-Paschoal-Esteves Lima, Fernando-Oliveira Costa

**Affiliations:** 1 Department of Dental Clinics, Oral Surgery and Oral Pathology, School of Dentistry, Federal University of Minas Gerais, Belo Horizonte, Minas Gerais, Brazil

## Abstract

**Background:**

Preemptive analgesia has as its basic principle the administration of analgesics before the onset of painful stimuli, in order to reduce or prevent postoperative pain, but this question is little explored in implantology. Thus, this study was conducted in order to evaluate the clinical efficacy of ibuprofen in pain prevention after unit implant surgery.

**Material and Methods:**

For this triple-blind, parallel, placebo-controlled and randomized clinical trial, 54 insertion surgeries of unitary implants were performed. Two groups have received two different protocols 1 hour before surgery: Ibuprofen group (IBU) 600 mg of ibuprofen; and (2) placebo group (maize starch). The intensity of the pain was evaluated through the visual analogue scale (VAS) in 6 times (1, 6, 12, 24, 48 and 72 hours after the surgery). Patients were instructed to take 750 mg of paracetamol as rescue medication, if necessary. The occurrence and the intensity of pain were analyzed by means of an analysis of variance ANOVA with repeated measurements using the general linear model procedure.

**Results:**

The IBU group had significantly lower VAS scores overall (IBU = 0.30, ± 0.57; placebo = 1.14, ± 1.07; *p*<0.001) and at all times in the intra, intergroup comparisons and time/group interaction than the placebo group (*p*<0.001). The use of rescue medication was significantly lower and the postoperative time was longer in the IBU group compared to placebo (*p* = 0.002).

**Conclusions:**

The single use of ibuprofen was found to be significantly superior in reducing pain after unit implant surgery compared to placebo.

** Key words:**Analgesia, dental implants, pain, surgery, oral.

## Introduction

Pain is a factor hardly dissociated from dental treatment (1). Their presence or only the possibility of feeling it can trigger innumerable reactions such as fear and anxiety, which can directly interfere with the safety of looking for a particular treatment (2-4). In addition, the dental procedures that generate the greatest fear associated with the possibility of pain include surgical procedures, particularly those of implantology (1).

The concept of preemptive analgesia (PA) to reduce the postoperative pain was based on a series of experimental animal studies (5), which demonstrated central nervous system plasticity and sensitization after nociception. The PA has as basic principle the administration of analgesics before the onset of painful stimuli, in order to reduce or prevent postoperative pain (hyperalgesia), as well as to reduce the analgesic dose required in the postoperative period when compared to the dose used alone, after the occurrence of the pain stimulus (1).

Three classes of analgesic drugs [blockages with local anesthetics, non-steroidal anti-inflammatory drugs (NSAIDs) and opioids] have been used for this purpose alone or in combination (5,6).

Several studies have been conducted in PA, particularly in third molar extraction surgeries (7-12,) periodontal surgeries (13-15), and few studies in implant dentistry (1,16-18), with conflicting results. In addition, different types of drugs have been tested in PA (1,9,13-16) however without a consensual definition of the best protocol regarding the drug, posology and period of exposure to the drug.

Studies have shown a high preference for the use of Ibuprofen in pain control related to dental problems or dental procedures (12,16,19) Ibuprofen, a name derived from the initials of isobutylphenyl propionic acid, is a drug in the group of NSAIDs which acts by non-selectively inhibiting cyclooxygenase 1 and 2 thus avoiding the consequent formation of pro-inflammatory mediators by the arachidonic acid cascade (19).

In this sense, the hypothesis for this study was that individuals who receive PA with the use of ibuprofen (600 mg, oral) 1 hour before the implant surgery present lower postoperative pain in relation to individuals receiving placebo medication.

Thus, the objective of this study was to evaluate and to compare by means of a randomized clinical trial, parallel-controlled and triple blind the efficacy of preemptive administration of Ibuprofen (600 mg oral) 1 hour before surgery for single insertion of implants osseointegrated in the control of postoperative pain.

## Material and Methods

-Study sample

This study was conducted from November 2018 to May 2019, in a private clinic of Implantology in the city of Belo Horizonte, Brazil. Fifty-four eligible individuals, both genders, non-smokers, aged 37 to 74 years were selected to participate in this randomized clinical trial, controlled, parallel and triple blind.

The nature of the study was previously explained to each patient, who signed a consent form, and this study was approved by the Ethics Committee in Human Research of the Federal University of Minas Gerais, Brazil (83534618.5.0000.5149) and registered in Brazilian registry of clinical trials (ReBEC) under identifier RBR-4B5DSG (www.ensaiosclinicos.gov.br).

The following inclusion criteria were adopted: 1) good systemic health status (ASA I or II); 2) absence of current pain or presence of any oral inflammatory process; 3) without analgesics in the 3 weeks prior to the study; 4) lack of continuous use of steroid and non-steroidal anti-inflammatory drugs; 5) need to install unitary implants not concomitant with maxillary and mandibular exodontia; 6) implants of 3.75 mm in diameter with 11 to 13 mm in length; 7) presence of compatible bone width and height determined by tomographic examination (cone-bean computed tomography). The following were excluded: pregnant or lactating women, use of some type of medication that could affect the perception of pain, history of allergy or intolerance to the drugs (ibuprofen and paracetamol) used in the research; and history of alcohol or drug abuse.

-Sample Calculation

Using statistical power analysis software R (software, R Foundation, Vienna, Austria), it was determined that 25 patients per group would be required to achieve 80% of power at 95% of confidence interval (CI), assuming moderate to intense changes in pain score 24 hours after surgery in at least one group and non-normal distribution data (16). In order to compensate for possible data loss during the study, the number of individuals allocated per group was increased by 10%, with 27 patients per group.

Randomization

**Figure 1:**
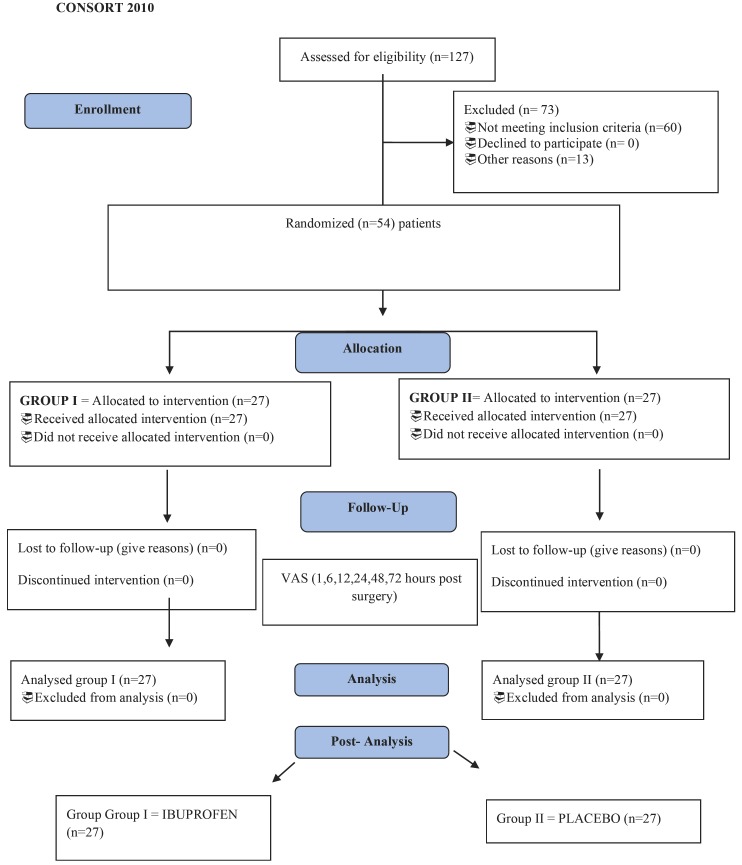
Flow Diagram.

All patients involved in this study were selected through a sequential non-probability method, that is, consecutive cases were drawn for each eligible intervention using, alternatively, envelopes denominated group I and II in consultation prior to surgery. Randomization in the groups was performed by a sequential stratified randomization process that consisted of the use of 54 opaque envelopes, where the identifications of the treatment groups (groups I and II) were placed. The envelopes were sealed and scrambled, and later numbered in sequential order. For each new entrant in the study, a subsequent numbering envelope was opened. The flow diagram is show in Figure 1.

-Medications used

The following drugs were used in this research: Ibuprofen(Alivium ® Mantecorp Industry, São Paulo, Brazil) – Tablets 600 mg, a placebo containing maize starch and Paracetamol(Tylenol ® - Johnson & Johnson, São José dos Campos, Brazil) - Tablets 750 mg. All drugs used in the research were conditioned in white capsules, placed in bottles identified as group I or II medication by the researcher (G.M.P).

-Instructions for the study and collection of variables

All participants in consultation prior to surgery have received detailed instructions for the correct form filling and visual analogue scale (VAS) for postoperative pain assessment. They were advised to contact the operator in case of any doubt. Thus, following randomization procedures, the individuals received a vial containing a medicine capsule from Group I or a medicine capsule from Group II, which should be ingested 1 hour before the surgical procedure. In addition, Paracetamol 750 mg Tablets were made available to all individuals to be used as a rescue medication in case of pain. The use of rescue medication was at the discretion of the patient in case of pain. It is noteworthy that all patients registered in this consultation on mobile phones a reminder to take the drug 60 minutes before the scheduled time for surgery.

The following information was collected in this consultation: age, gender, body mass index, educational level, family income expressed in minimum salaries, as well as additional information on health in general.

-Visual Analog Scale (VAS)

Participants have completed a visual analogue scale (VAS), where they indicated the presence and intensity of pain (20,21). This scale has 10 cm of lenght, subdivided into five equal parts, where one end corresponds to no pain (0), and the other end to severe pain (10,22). The registry was completed by all participants at the following times: 1, 6, 12, 24, 48 and 72 hours post-surgery. In addition, in case of the necessity of the use of rescue medication, the registration was made at the time of taking the medication in relation to the time elapsed after the end of the surgery.

-Surgical Procedures

Surgical procedures were performed by a single operator (F.O.C.) through a mucoperiosteal flap where unitary implants (Titamax, external hexagon, Neodent, Paraná, Brazil) were inserted according to the manufacturer’s instruction. The individuals were anesthetized with a local infiltrative anesthetic (Prilocaine hydrochloride 30 mg/ml) block using 2 to 4 anesthetic tubes at most. All surgical procedures were performed in up to 1 hour.

-Statistical analysis

The analyzes were performed blindly by a researcher (LOMC) continuing to assign the groups as groups I and II. The variables of interest were reported for descriptive measures (mean, standard deviation and CI) and analyzed using the chi-square test and t-test when appropriate. The perception of pain over time (VAS 1 hour to 72 hours) between the groups (Placebo vs IBU) was analyzed separately by the Friedmam test (analysis of variance by ranks) and subsequently by means of a 2-way analysis of variance ANOVA with measures repeated through general linear model procedure (GLM). Comparative analysis of the need for medication rescue for pain control and survival analysis for need of rescue were performed using the Manny-Whitney and Mantel-Cox tests, respectivaly. The data were analyzed using the statistical program (R software). Subsequently the analysis according to the initial knowledgeable investigator (G.M.P.) of the groups has revealed that group I used Ibuprofen (group IBU) and group II the capsule with maize starch (placebo group).

## Results

The study has involved 54 non-smoking patients of both genders, with no significant differences in BMI, surgery time, anatomical position of the implants, socioeconomic and cultural level among the IBU and placebo groups ( Table 1). However, they have presented significant differences for age and gender, the IBU group consisted of 12 women and 15 men and the placebo group of 21 women and 6 men (*p* = 0.012). The mean age in the IBU group was 61.07 ± 8.01, while in the control group 55.63 ± 9.36 (*p* = 0.026) ( Table 1). No major adverse effects (infections, major edemas or bleeding) and no side effects of the drugs were reported in both groups.

**Table 1:**
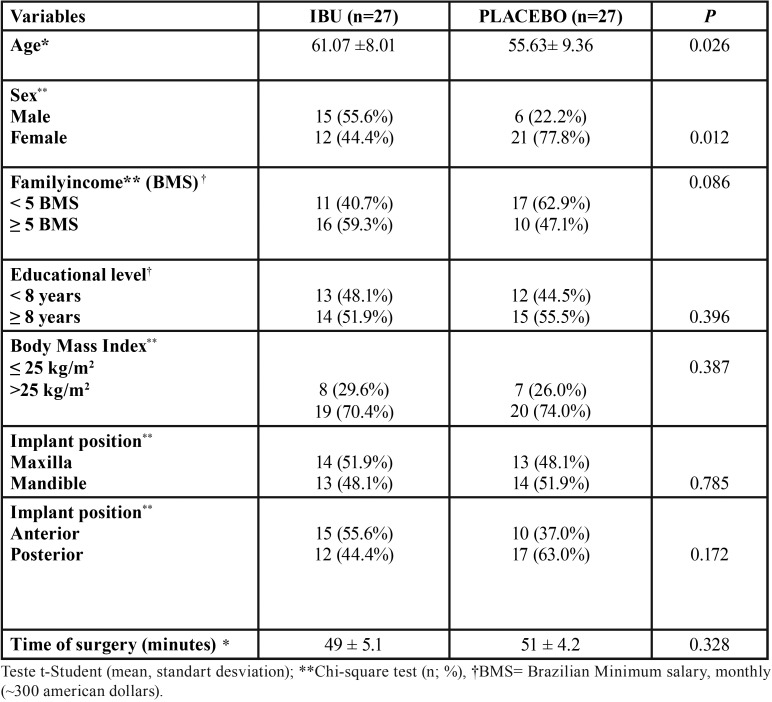
Variables of interest*.

**Figure 2:**
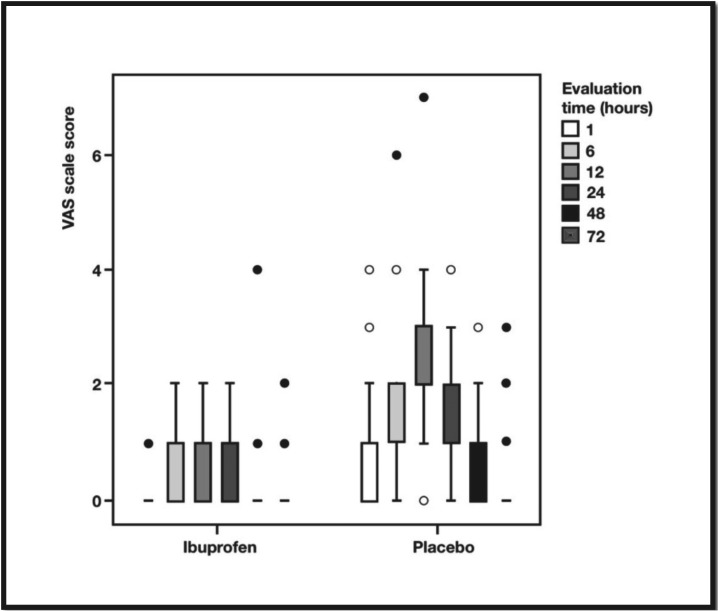
Boxplot distribuition VAS scores in time by groups.

 Table 2 and Figure 2 shows the comparisons of the scores obtained by the VAS for pain according to the groups and times. Overall, the IBU group (VAS = 0.30, ± 0.57) had significantly lower VAS scores than the placebo group (VAS = 1.14, ± 1.07) (*p*<0.001). In addition, the IBU group also presented values significantly lower than those reported in the control group at all times in the intragroup, intergroup comparisons and in time and group interaction (*p* <0.001).

**Table 2:**
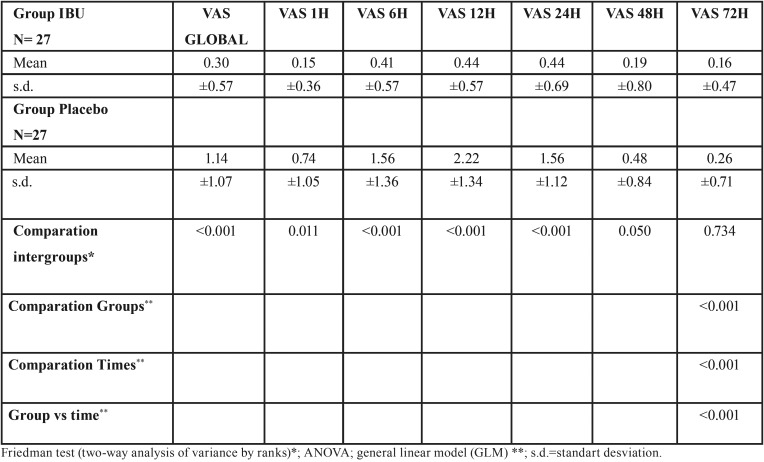
Comparison of the VAS variable for pain according to groups and times.

Regarding the need to use of the rescue medication to control postoperative pain, the IBU group has used significantly less rescue than that observed in the placebo group ( Table 3; Placebo> IBU; *p* = 0.002).

**Table 3:**
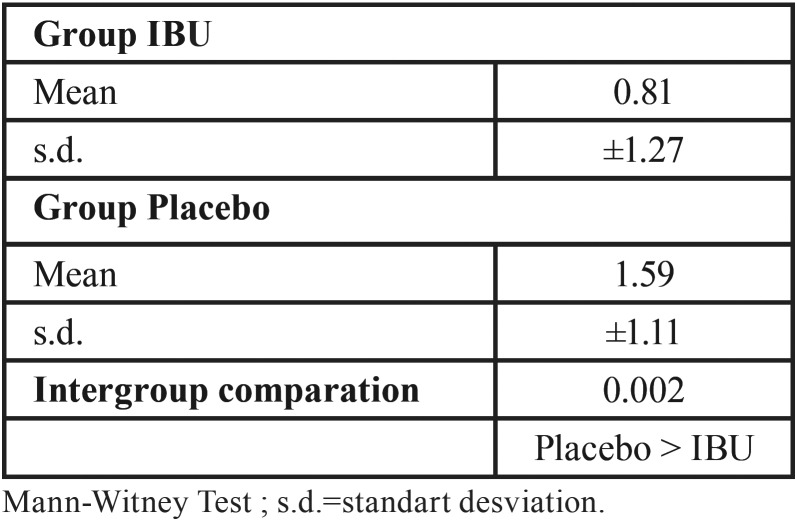
Comparative analysis of the need for medication rescue for pain control.

**Figure 3:**
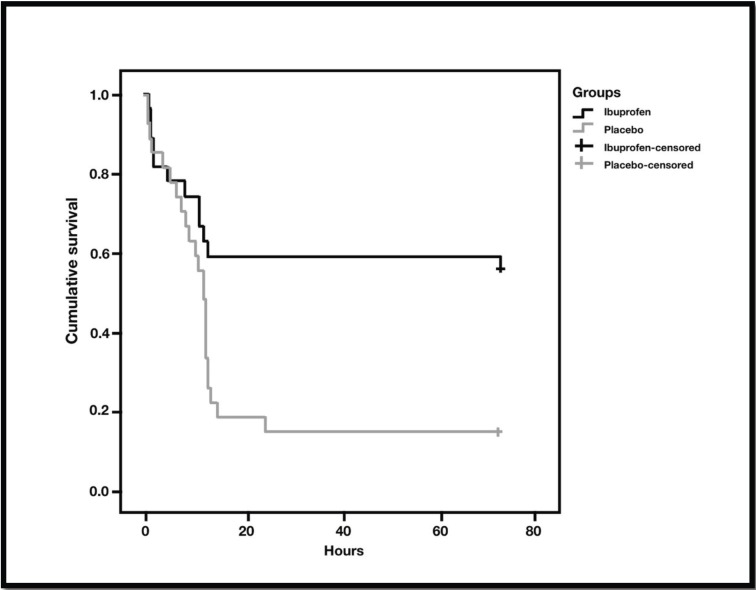
Survival analysis for need of rescue.

Corroborating these findings,  Table 4 and Figure 3 present a survival analysis of the need to use of the rescue medication, i.e., time elapsed from the first rescue event (mean in hours). In the placebo group the elapsed time was significantly lower (18.85 ± 4.37 hours) in relation to the IBU group (45.24, ± 6.51 hours, *p* = 0.007).

**Table 4:**
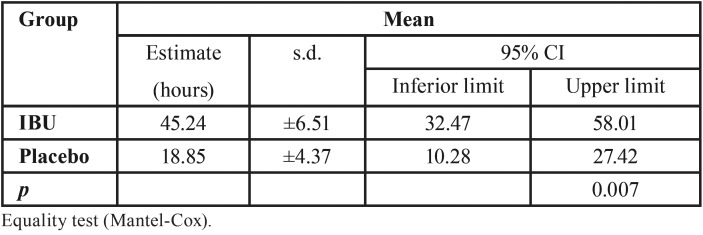
Survival analysis for need of rescue.

## Discussion

In this study, the use of ibuprofen 1 hour prior to the surgical procedures for insertion of unitary implants has revealed, overall, a significant effect on reduction of postoperative pain, at all times evaluated, less need for rescue medication, as well as a longer time for the occurrence of the rescue event in relation to the individuals with the use of placebo medication. Thus our hypothesis of its beneficial effect on the reduction of postoperative pain was affirmative.

Theoretically, PA is defined as an antinociceptive treatment (5), which aims to prevent central and peripheral sensitization, reducing or preventing the amplification of postoperative pain. It is believed that this strategy guarantees a reduction in the consumption of analgesics in the postoperative period, providing comfort and reducing the recovery time of the patient (5,23).

In our study, the IBU and placebo groups differed significantly in age and gender, without differences in relation to the other variables studied, such as anatomical position of the implants, surgery time, BMI, family income and socioeconomic level.

Analyzing the plurality of factors that may interfere with perceived pain (4), the absence of differences in almost all variables surveyed between the groups in our study seems to contribute to the validation of our findings. It is known that the level of pain correlated with physical stimuli as a result of dental interventions is difficult to determine, since perceived pain depends on an individual threshold and is influenced by emotional, cognitive and cultural factors (24-27).

In a review about orofacial pain (25) the data were not conclusive about the role of gender and age influencing pain. It has been reported that women are more willing to report pain than men, and that this perception appears to be associated at the pain threshold with respect to heat tolerance. However, after controlling the willingness to report pain, the difference in the thermal pain threshold was no longer statistically significant. In addition, a study on predictors of pain in dental treatment has reported no differences in pain reported in relation to gender and age (24).

Future research needs to elucidate the influence of socio cultural, environmental, and psychological factors on pain as well as the effects of sex on factors that protect against the development of pain or that prevent pain from becoming debilitating (28). Thus, we consider that due to the randomization process and the conflicting pain data regarding gender and age, we do not believe in the interference of this variable in our results.

Several studies have been conducted in PA in dentistry. Most studies focus on third molar extraction surgeries (7-10,12,28) followed by periodontal surgeries (13-15) and studies on implantology (1,16-18).

Moreover, in almost all different procedures using PA, the results are conflicting. In addition, studies with different types of drugs have been tested in PA but without a consensual definition of the best protocol for the drug, posology and period of exposure to the drug. Thus, there is a tendency in PA to test steroid and non-steroidal anti-inflammatory drugs with only one study testing the use of ibuprofen.

In a systematic review (29) it was verified that several clinical trials were conducted seeking to prove the efficacy of PA in the clinical dental practice, however, they exhibited controversial results. According to Kissin (3) it is not possible to detect a preemptive effect in comparative studies of groups in which the analgesic is administered only in the pre-incisional and post-incisional period, due to the complexity in the central sensitization mechanisms and the technical difficulties for the studies, considering it necessary the pre, trans and postoperative analgesic regimen. However, exploring its effects at different time intervals may bring further clarification of its effectiveness. In this context, this study has searched for this information in periods ranging from 1 to 72 hours, as well as the additional need for rescue medication for pain control.

Additionally, in our findings, ibuprofen has proven to be very effective in reducing pain in the immediate postoperative period from 1 to 6 hours compared to placebo, significantly reducing the need for rescue medication and increasing the time required for rescue medication, thus according to the theoretical foundations of PA this initial reduction is very important because it has a positive effect on reducing pain at subsequent intervals of time (1,5,23).

In this context, our finding is supported by a systematic review (29) of the oral kinetics of oral ibuprofen (30 studies evaluating 1,015 individuals) which showed that mean maximum plasma concentrations of fast acting formulations occurred within 50 minutes (29-35 minutes for arginine, lysine and sodium salts) compared to 90 minutes for standard formulations. Initial rapid reduction of pain intensity was also associated with reduced need for remedies. Additionally, the ibuprofen by inhibiting the production of prostaglandins, it leaves the gastric mucosa less protected against acidity, being its therapeutic efficacy easily seen overcoming the severity of its side effects (19).

Another point to be discussed refers to the extent and numbers of implants inserted in the surgical procedure. Low pain scores are related to implant surgeries and according to Kim *et al.* (27) pain and anxiety scores are proportional to the number of implants inserted and the length of the surgical procedure. However, regarding these questions, our study presents the advantages of standardization of insertion of only one implant, with a single operator and similar surgery time between the groups.

The present study may be considered as a good starting point for future studies aimed at providing additional information on the use of ibuprofen in PA in implant surgeries. However, prospective multicentric studies, in different populations, with different surgical and dosing protocols, are necessary to confirm these findings and to establish a beneficial protocol for the control of pain in dental implant surgeries.

The present study demonstrated that the use of ibuprofen 1 hour before to the surgical procedures for insertion of unitary implants has revealed a significant effect on the reduction of postoperative pain overall at all evaluated times has shown less need for the use of rescue medication and, when necessary, the time of use has been shorter when compared to individuals on placebo. Thus, its use can be considered a beneficial adjuvant for the control of postoperative pain in dental implant surgeries.
